# Endogenous polyamine function—the RNA perspective

**DOI:** 10.1093/nar/gku837

**Published:** 2014-09-17

**Authors:** Helen L. Lightfoot, Jonathan Hall

**Affiliations:** Department of Chemistry and Applied Biosciences, Institute of Pharmaceutical Sciences, ETH Zürich, CH-8093, Zürich, Switzerland

## Abstract

Recent progress with techniques for monitoring RNA structure in cells such as ‘DMS-Seq’ and ‘Structure-Seq’ suggests that a new era of RNA structure-function exploration is on the horizon. This will also include systematic investigation of the factors required for the structural integrity of RNA. In this context, much evidence accumulated over 50 years suggests that polyamines play important roles as modulators of RNA structure. Here, we summarize and discuss recent literature relating to the roles of these small endogenous molecules in RNA function. We have included studies directed at understanding the binding interactions of polyamines with polynucleotides, tRNA, rRNA, mRNA and ribozymes using chemical, biochemical and spectroscopic tools. In brief, polyamines bind RNA in a sequence-selective fashion and induce changes in RNA structure in context-dependent manners. In some cases the functional consequences of these interactions have been observed in cells. Most notably, polyamine-mediated effects on RNA are frequently distinct from those of divalent cations (i.e. Mg^2+^) confirming their roles as independent molecular entities which help drive RNA-mediated processes.

## INTRODUCTION

### Discovery and physical properties of polyamines

**Figure 1.**
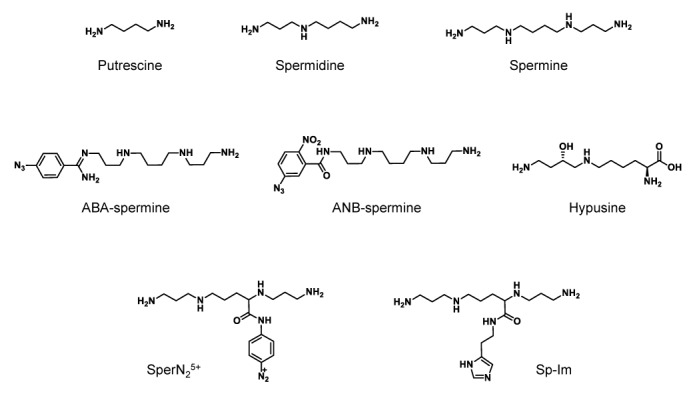
Chemical structures of the polyamines and polyamine analogues discussed throughout this review.

The polyamine spermine (Figure 1) is a linear aliphatic nitrogenous base which was first isolated as the phosphate salt from human semen in 1678. In the 1920s Dudley and Rosenheim finally extracted the free base spermine, noting its ‘characteristic odor’ and proposed the chemical formula NH_2_(CH_2_)_3_NH(CH_2_)_4_NH(CH_2_)_3_NH_2_. The isolation and characterization of a second polyamine, spermidine was described shortly thereafter (Figure 1). For an excellent historical perspective on the identification and early characterization of the polyamines, including the diamine putrescine (Figure 1), the reader is referred to the review by Tabor and Tabor ((1) and references therein).

The conformations of free polyamines have been monitored using a variety of techniques including X-ray crystallography, nuclear magnetic resonance (NMR), Ramon, Inelastic Neutron Scattering spectroscopies and molecular modeling (2–5). In these studies, polyamines were reported to adopt a variety of conformations including linear all-trans conformations, gauche twisted conformations and cyclic conformations. Taking these works together, it appears likely that the predominant conformations of polyamines depend upon a balance between stabilizing intramolecular H-bonds and minimization of repulsive steric and electrostatic interactions and that these are strongly influenced by the nature and pH of the medium (2). Titration studies monitoring pKa's of spermine and spermidine suggest at physiological pH all polyamine nitrogens are probably mainly in the protonated state, which is likely significant not only for their conformation but also their properties in cells (6,7).

### Distribution of polyamines in cells

Polyamines are thought to be biosynthesized ubiquitously (8) with the overall (free and bound) cellular concentrations estimated to be in the millimolar range (9,10). Various organisms produce varying amounts of polyamines. For example, *Escherichia coli* and *Bacillus stearothermophilus* predominantly synthesize putrescine and spermidine, and spermidine and spermine, respectively (11). On the other hand, mammalian cells produce putrescine, spermidine and spermine (8). Until now, our knowledge of the cellular distribution of polyamines has derived from: (i) binding studies of polyamine and subcellular components *in vitro* (9,12–14), (ii) detection of polyamines in isolated cellular macromolecules (14–19), (iii) fluorescence-based cytochemical studies (20–22), (iv) experiments using autoradiographic localization (18,21,23,24) and (v) immunohistochemistry (20,21,25–32). Technical issues abound with all of these methods (1,33). In particular, the facile redistribution and displacement of polyamines in cells has rendered insights derived from isolated cellular components difficult to extrapolate to *in vivo* settings (34–36). In two prominent papers, Watanebe *et* *al*. and Miyamoto *et* *al*. estimated the cellular distribution of polyamines and the diamine, putrescine using *in vitro* based macromolecule-polyamine binding studies (12,13,36). They found that in mammalian and *E. Coli* cells, the polyamines spermidine and spermine probably exist predominately as polyamine–RNA complexes, with 1–4 equivalents of polyamine bound per 100 equivalents of RNA phosphates. In contrast, putrescine was distributed approximately equally between RNA and its free form, in agreement with a previous report (14). Notably, neither polyamines nor putrescine were found to bind to cytoplasmic proteins. Other independent studies using alternative techniques have described similar findings. For example, immunohistochemistry utilizing an antibody targeting spermine/spermidine (ASPM-29) as well as ^13^C-NMR studies of isotopically labeled spermidine both indicated that polyamines predominantly co-locate with RNA or the ribosomal fraction (10,19,28,31,32,37).

## Polyamine functions

Polyamines play many roles in a wide variety of organisms (reviewed in (36,38–51)). For example, in mammals, polyamines function in diverse physiological processes including immunity, aging, hair growth and wound healing. Accordingly, the cellular concentrations of polyamines reflect these functions and vary widely according to cell type and context. In terms of cellular mechanisms, polyamines play important roles in messenger RNA (mRNA) translation and stability, both in a global sense as well as in specific cases. In addition, they are reported to modulate kinase activities, small RNA methylation, transcriptional regulation, microtubule assembly and ion channel regulation. Through a unique mechanism, spermidine also acts as precursor of hypusine (Figure 1), a post-translationally modified amino acid of the initiation factor elF5a in protein synthesis (52). Numerous works have described how polyamines regulate their own homeostasis, using some of the aforementioned mechanisms (reviewed in (42,53–56)). In spite of the multitude and diversity of processes which polyamines are known to influence, mechanistic insight into how they perform these functions is usually sparse.

Polyamines function through direct and indirect influences on the structure and stability of a variety of macromolecules. For example, polyamines are known to directly bind and in some cases stabilize and/or induce transitions between B-form, Z-form, A-form, triplex and quadruplex-form DNA. In these cases, polyamine-DNA binding selectivity for G–C-rich major grooves, A–T homo-dimers, as well as A, A–T and Pu-Py tracts has been reported (57–66). Indirectly, polyamines can function as scavengers of oxygen free radicals, and consequently protect nucleic acid and other cellular components from oxidative damage (67). Of note, oxidation-driven polyamine catabolism acts as a source of hydrogen peroxide, a precursor for reactive oxygen species, as well as reactive aldehydes both of which are capable of damaging cellular components (55,67). This suggests that the cellular damage resulting from the depletion of polyamines is escalated by the evolving toxic products of the oxidase responsible for their depletion (67).

## POLYAMINE–RNA INTERACTIONS AT THE MOLECULAR LEVEL

Numerous works over the past 50 years have suggested that polyamines function as modulators of RNA structure. A handful of reviews in the 1960s, 1970s and 1980s have covered this extensive field in detail (1,18,33) and their focus has traditionally been directed at the interactions between polyamines and transfer RNA (tRNA) or synthetic RNA models. In the last two decades, however, numerous new studies have appeared concentrated on understanding the interactions and functional consequences of polyamines binding to additional diverse RNAs (e.g. ribosomal RNA (rRNA), ribozyme, mRNA, small nuclear RNA (snRNA) and viral RNA). Although a wide range of polyamine analogs are biosynthesized by a variety of organisms (67), here we have focused our literature survey on the dominantly investigated, spermidine and spermine.

### Synthetic RNA models

Experiments performed with polyamines and model RNAs have provided key information about the structural and functional consequences of polyamine–RNA interactions in cells. Polyamines are well known to precipitate short RNA sequences and to increase the melting temperatures of RNA duplexes (1,68–71). In this vein, short RNA model sequences, e.g. polyU, polyA, polyC and polyI, as well as their double-stranded counterparts, have often been used to investigate the sequence dependence of the polyamine–RNA interaction. For single-stranded RNA, Huang and Felsenfeld and Ikemura employed precipitation (70) and gel filtration techniques (72), respectively, to learn that spermine exhibits a preference for polyA and polyI over polyC and polyU. In contrast, other groups have reported that spermine prefers to associate with RNAs in the order of polyU > polyC and polyA (73,74), and that spermidine interacts most preferably with polyGC RNA (12). The effects of polyamines on polyU structure were investigated in detail by Szer *et al.*, who found that spermidine and spermine are more effective than either diamines or magnesium at raising the melting temperature of polyU. These observations indicated that spermidine and spermine stabilize RNA structure (75). In a subsequent study, the pH dependence of the optical rotary dispersion of the spermine-polyU complex was examined. It was found that at pH 8.5 spermine does not influence the rotary dispersion of the complex. However, at pH 7 and pH 5.5 cotton effects were seen, suggesting that under more acidic conditions spermine increases base stacking in the polyU sequence (76). Thrierr *et al.* (77) confirmed polyamine-mediated base stacking through absorbance and circular dichroism (CD) measurements; from light scattering studies they concluded that intramolecular rather than intermolecular associations in the polyU were responsible for the effects. Efforts to rationalize the data focused on the structural properties of the model RNAs. Whereas polyA and polyC, as well as the triple-stranded complexes of polyI form coils, polyU does not display any ordered structure (72). Hence, spermine and spermidine were hypothesized to specifically stabilize a polyU structure by inducing base-stacking through polyU intramolecular associations (75–77). However, they did not elaborate on the nature of these interactions. An investigation into the effects of various amines on the resonance of protons in single stranded polyA, polyU and polyC by ^1^H-NMR spectroscopy found that spermine and spermidine selectively, and charge-dependently, eliminated the resonance of the 2′-OH protons at ratios of about one spermine per ten phosphates. The lack of effects on mononucleosides suggested that the phosphate backbone is required for the spermine-RNA interaction. Thus, the authors proposed a model in which the polyamine is H-bonded to both the phosphate and the 2′-OH, displacing a bound water molecule, permitting rapid exchange of the 2′-OH proton (78).

The binding of spermine is stronger to double-stranded than to single-stranded polynucleotides (73). It also shows a clear sequence-dependent precipitation of poly(A+U) in contrast to poly(C+I) (70), demonstrating again that oligonucleotide sequence is a key directive of the polyamine–RNA interaction. A slight preference of spermine and spermidine for poly(A+U) over poly(G+C) was later confirmed independently by gel filtration assays (73,74).

One of the few well-characterized functional consequences of polyamine-oligoribonucleotide (ORN) interactions concerns the non-enzymatic hydrolysis of ORNs (79–81). The cleavage reaction of single-stranded RNA was seen to depend upon several factors including polyamine structure, its concentration, the ORN sequence and the pH (81,82). The authors proposed a mechanism in which an intramolecular acid-base cooperation of the polyamine similar to that of two imidazoles in ribonucleases enabled the nucleophilic attack of the ribose 2′-OH at phosphorus (82).

### mRNA and snRNA

The ‘polyamine modulon’ is a set of genes whose expression is enhanced by polyamines at the level of translation (36,83,84). Polyamine modulons have been identified in yeast, mammalian cells and *E. coli* (83,85–88). In the majority of cases, the mechanisms underlying polyamine-mediated stimulation of translation are unknown, however, several hypotheses have been forwarded (36,83). These include: enhancement of translation initiation at inefﬁcient codons, e.g. for Cya, Cra, SpoT, UvrY, RRF in *E. coli*; changes in the position of an obscure or distant Shine-Dalgarno (SD) sequence, e.g. for FecI, Fis, RpoN, H-NS RpoZ, OppA, RMF and CpxR; stimulation of read through of an amber codon (RpoS) and stimulation of a plus-one (+1) frame-shift (PrfB) (83,87,89–92). In mammalian cells and yeast, attention has predominantly focused on the influence of polyamines on ribosomal shuttling, e.g. for Cox4 and Cct2 (85,86). It is conceivable that in some of these cases direct interactions between the polyamine and the mRNAs are responsible. This implies that polyamines are ligands for riboswitches in the genes of the polyamine modulon. Several riboswitch elements have been characterized in bacteria for which a wide variety of ligand structures are known (e.g. lysine, Mg^2+^, F^−^ and S-adenosylmethionine) (93). However, polyamines have not been demonstrated to perform such a role, despite strong circumstantial evidence outlined below.

**Figure 2.**
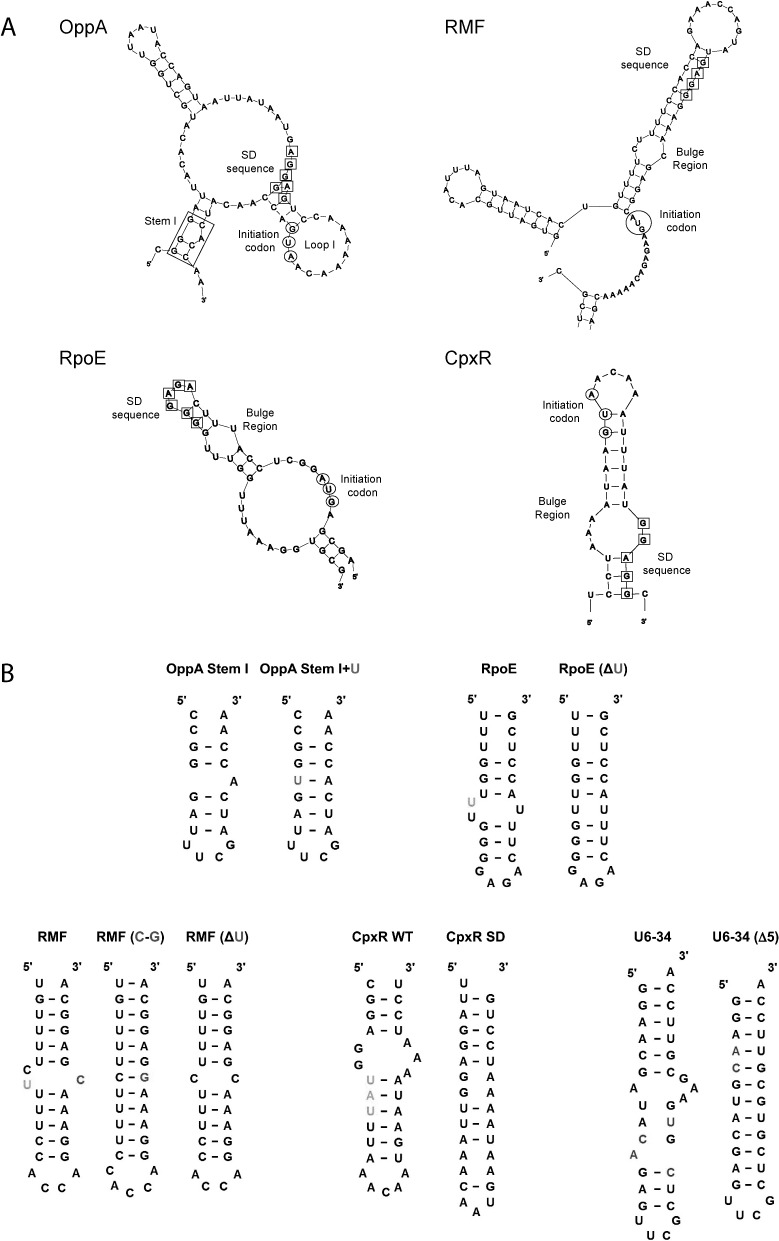
(**A**) Predicted structures of the initiation regions of OppA, RMF, RpoE and CpxR (only relevant sections shown). The nucleotide residues of the SD sequences are boxed and those of the initiation codon (AUG) circled. Stem I of OppA is boxed. (**B**) Mutants of the bulged stems used in the overexpression or biophysical studies. Highlighted: green—nucleotide (nt) deletion; blue—nt addition; red—nt mutated; purple—nt conformational changes.

Polyamine-mediated stimulation of translation has been investigated for several members of the *E. coli* modulon (OppA, RMF, cpxR, rpoE) where their SD sequences are all obscure or distant from their initiation codon (AUG) (36,90,92,94–96). For example, enzymatic structural probing of the OppA mRNA initiation region (Figure 2A) revealed several effects of spermidine binding. These included a structural relaxation at the SD and AUG sites accompanied by stacking of the predicted loop structure (Loop I) between these elements. In addition, a stabilization of a predicted A-bulged, GC-rich stem (Stem I) situated close to the AUG and SD sites was observed. The probable consequences of these structural alterations are an increased proximity and an enhanced exposure of the SD and AUG sites, both of which would be expected to facilitate translation initiation (94). The importance of Stem I for the effect of spermidine on translation had previously been shown in mRNA fragment deletion studies (97). Notably, the introduction of destabilizing mutations in Stem I of OppA mRNA inhibited the positive effect of spermidine on stimulation of tRNA-ribosome binding (94). On the other hand, replacement of Stem I with a fully complementary mutant (Stem I+U) (Figure 2B) led to a decrease in polyamine-mediated enhancement of OppA translation (5.1-fold to 1.5-fold). This clearly suggested that the A-bulge of Stem I plays a direct role in spermidine's effect on OppA translation. Supporting evidence for this was provided by measurements of CD spectra of both stems, under a variety of conditions. These showed that spermidine (but not Mg^2+^) binds selectively to the A-bulge region and stabilizes the A-form helix of Stem I, but not of Stem I+U (96). Taken together, this suggests that spermidine binds and stabilizes an A-bulged, GC-rich stem in the initiation region of OppA mRNA. This interaction possibly re-positions or stabilizes the SD sequence at an optimal distance from the AUG or it increases the accessibility of the SD and AUG sequences, both of which lead to more efficient translation initiation.

Similar bulged-out stems, in the initiation regions of RMF, RpoE and CpxR (Figure 2A) were later also suggested to contribute to an enhanced stimulation of translation by polyamines through the re-positioning of SD sequences. For example, shifting the SD sequence away from its normal position reduced the effects of polyamines on RMF and RpoE translation. Furthermore, mutations and deletions of bulged pyrimidines in the U-rich stems linking the SD and AUG sequences of both mRNAs (Figure 2B) also reduced the intensity of polyamine stimulation of translation (90,95). Using CD it could be seen that spermidine selectively stabilized the U-rich, A-form structure of RMF mRNA in a manner distinct from that of Mg^2+^ and putrescine (90). Similar observations were made in the case of RpoE mRNA. Spermidine stabilizes the U-rich A-form helix and enhances RNA-dependent tRNA-ribosomal binding and translation, in manners which are bulge- and temperature-dependent (95). In the case of CpxR, stimulation of translation by polyamines in the presence of Cu^2+^—a metal required for CpxR-mediated transcriptional modulation of several *E. coli* genes—is dependent on the distance between SD and AUG sequences positioned at each end of a predicted bulged A-U rich stem (Figure 2A). Spermidine in the presence of Cu^2+^ selectively stabilizes the bulged stem in the initiation region of CpxR (CpxR WT: Figure 2B) and causes a re-positioning of the normally distal SD sequence, leading to increased translation (92). A mutant CpxR (CpxR SD; Figure 2B) RNA where the SD is re-located was not selectively stabilized.

The effects of spermidine on bulged-out regions of other double-stranded RNAs lend further support to these postulated mechanisms. In one study the binding site for influenza virus type A NS1 protein on the non-coding snRNA U6 was investigated (98). An NMR structure of this subsequence of the U6 snRNA (U6–34: Figure 2B) has been resolved (99). Using U6–34 and a mutant bearing a fully complementary stem (U6–34 (Δ5)) as a control, the effects of spermidine on the conformation of the bulged nucleotides in the presence of Mg^2+^ and K^+^ were examined by NMR. In the presence of spermidine-specific alternations of an adenine, two cytosines and an uracil in a bulged-out region of U6–34 were observed. On the other hand, nucleotides in the second bulged region of U6–34 were not significantly affected suggesting that spermidine interacts with the RNA in a specific fashion. In contrast, spermidine only induced changes in U6–34 (Δ5) at nucleotides positioned in the common double-stranded regions of U6–34 and U6–34(Δ5). Additional evidence came from CD spectra in which spermidine was shown to stabilize the A-form helix of U6–34 in a manner which Mg^2+^ could not. In contrast, the extent of helical stabilization for U6–34(Δ5) by spermidine was similar to that for Mg^2+^. This suggested that spermidine binds and stabilizes distinctly the bulge region in U6–34. Finally, binding of the NS1–2 peptide (a region of NS1) to U6–34 was inhibited selectively by spermidine, whereas the binding of NS1–2 to U6–34(Δ5) was inhibited by both Mg^2+^ and spermidine. These observations imply that the concentration of available cellular spermidine may be a decisive factor in the contribution of NS1 to inhibition of host translation by the virus (98).

Taking these studies together, it can be concluded that spermidine (but not Mg^2+^) selectively stabilizes functionally relevant A-form RNA helices with bulged-out regions. The evidence suggests that this interaction, for at least some mRNAs, contributes to spermidine's effect on translation. In these examples the bulged helical regions were mainly U-rich stems in the initiation regions of mRNAs. The importance of a high U-content for spermidine-mediated enhancement of polypeptide synthesis was previously noted in studies using *E. coli* and *B. thuringiensis* cell-free systems (100). Importantly, this enhancement could not be compensated by any amount of Mg^2+^ (100), even though spermidine and Mg^2+^ often bind similarly to double-stranded RNA (90,92,96,98). In addition to effects on mRNA translation, spermidine has been shown to inhibit RNA–protein interactions, potentially through induced conformational changes in certain RNA bulged stems. Based on a small number of examples (36,90,92,94–96), affected bulges bound by spermidine typically range from 1 to 5 nucleotides (nt) in size, and can be categorized into large purine-rich bulges (5 nt), medium-sized pyrimidine-rich bulges (3 nt) and single adenine bulges (1 nt). Additional examples are needed to determine whether this is a general trend.

### Bacterial rRNA

The ribosome is composed of two subunits, each a complex of proteins and rRNA. It is the primary site for protein synthesis in archaea, bacteria and eukarya. In bacteria, the complete 70S ribosome complex consists of 30S and 50S subunits. The 30S subunit is composed of 21 proteins and a 16S rRNA of ∼1500 nt. The larger 50S subunit is composed of 33 proteins, a 23S rRNA of ∼3000 nt and a 5S rRNA of ∼120 nt. Several complete crystallographic structures have been determined for *E. coli* and *T. Thermophilus* ribosomes, in addition to structures for individual ribosomal subunits (101). Key functional sites of the ribosome are (i) the peptidylation (P) site, (ii) the acceptor (A) site, (iii) the exit (E) site and (iv) the peptidyltransferase (PTase) catalytic site (101,102). Polyamines are well known to facilitate many ribosomal functions and also to aid ribosome crystallization (103).

#### General ribosomal-polyamine binding properties

Interactions of polyamines with rRNA have been investigated by several groups. Binding constants of polyamine–rRNA complexes has been reported to be 0.18 × 10^4^ M^−1^ for spermine and 2.2 × 10^3^ M^−1^ for spermidine (74), with the number of binding sites estimated to be 0.11 amines per phosphate of nucleic acid (NAP) for spermidine (74), and a relatively wide-ranging 0.082–0.133 NAP for spermine (9,10,74). The binding of spermine to rRNA, e.g. *B. stearothermophilus* rRNA was dependent on the ionic strength and pH of the solution, but independent of temperature over a range of 4–60°C (9,10,74).

#### Competition studies

Initial efforts to clarify the manner by which polyamines bind to rRNA explored the use of the intercalators acridine orange and ethidium bromide as competitor compounds. Binding activities were monitored by spectrophotometric or gel filtration-based methods (10,11,104). The results suggested that polyamines do indeed compete with intercalators to bind predominantly double-stranded regions in rRNA *in vitro*. However, incongruences in some studies were noted, particularly concerning competition for spermine binding sites in ribosomes. For example, in two independent studies, acridine orange was not capable of competing with spermine in *B. stearothermophilus* ribosomes (10,11). However, in a later investigation, ethidium bromide successfully displaced the majority of bound spermine (up to 80%) (104) in the same ribosome complex (10,11). For sites where spermine was resistant to competition with intercalators, it was assumed that the polyamine had a higher affinity, or alternatively, it was located in inaccessible regions, possibly protected by ribosomal proteins (10,11,104).

#### Cross-linking studies

Cross-linking and fixation procedures have proven useful during studies investigating the interactions of polyamines with *E. coli* rRNA and ribosomes (104–107). These *in vitro* approaches offer an important advantage over mutagenesis and competitor studies because the sites of binding can be inferred. A valuable chemical tool for cross-linking experiments is ABA-spermine (Figure 1), a photoreactive polyamine containing an azidobenzamidino group linked to the terminal position of spermine. ABA-spermine retains most of the biochemical properties of spermine, including its interactions with structured RNAs (108,109). Upon ultraviolet (UV) irradiation the arylazido group of ABA-spermine is converted to a short-lived nitrene. This reacts immediately with proximal groups, covalently linking ABA-spermine to RNA or protein. As the arylazido tether (ABA-) is 9 Å long, this places the cross-linking site within 1 nt of the terminal amino group of spermine. The cross-linked sites in the RNA can be identified through various methods, such as RNase H cleavage and primer extension analysis. Furthermore, the cross-linker can be competed away from its binding site by an excess of natural spermine during the cross-linking reactions. This technique has been used to map comprehensively the binding sites of spermine in rRNA, as well as rRNA in ribosomal complexes (105–107,110). The findings of these studies are summarized below.

**Figure 3.**
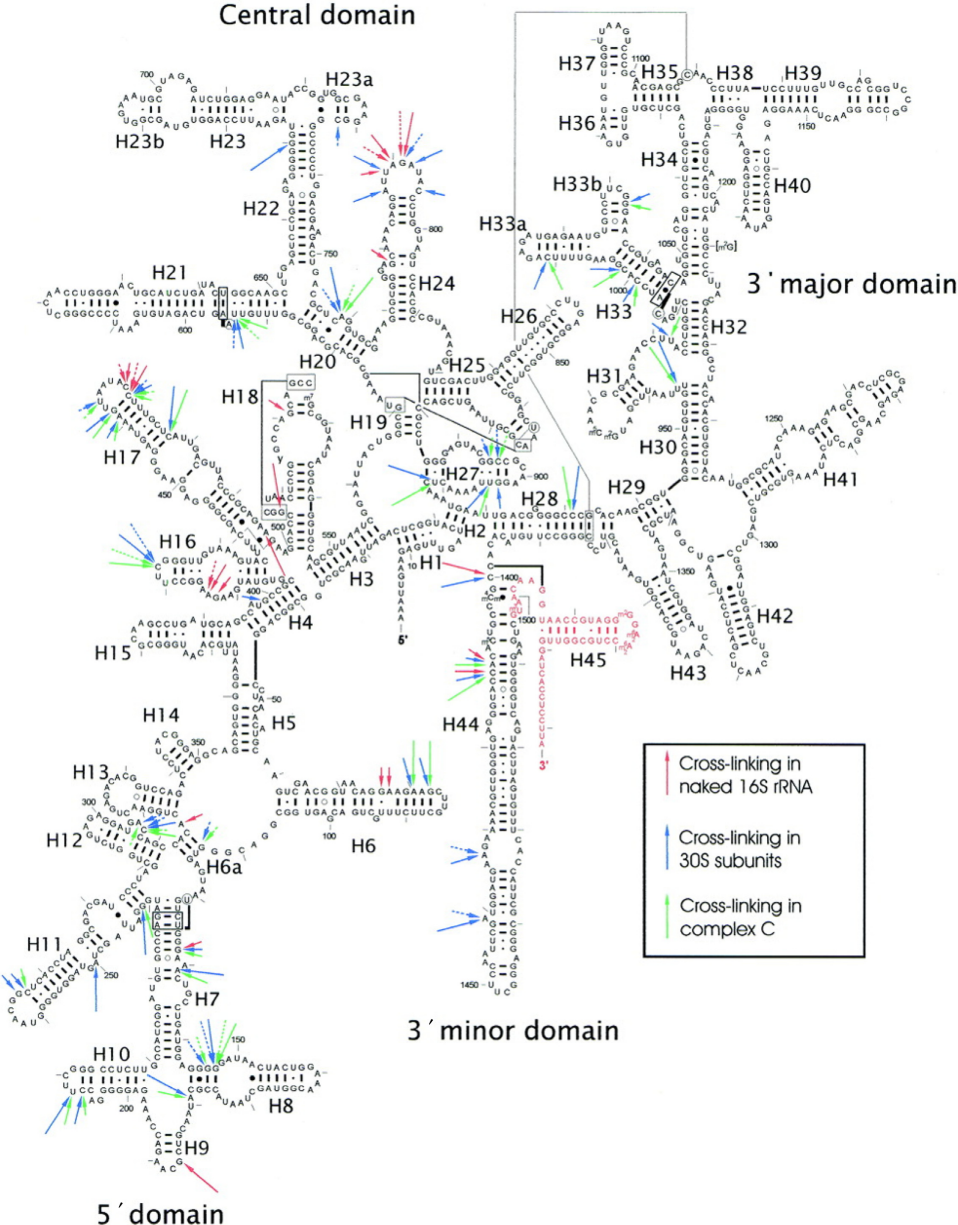
Summary of ABA-spermine cross-linking sites in 16S rRNA of *E. coli*. The arrows, discontinuous or solid, indicate sites labeling by 50 or 300 μM ABA-spermine, respectively. Long arrows—strong cross-links; medium arrows—intermediate cross-links; short arrows—weak cross-links. Nucleosides not analysed are shown in red. Figure taken from (106).

Spermine cross-linking sites were dispersed throughout the helices and loops of the 1542 nt 16S rRNA, with the exception of the 3′-major domain (Figure 3) (106). A total of 24 cross-linked sites, grouped into 13 independent regions were observed. Most of these were in, or directly adjacent to single-stranded regions. In the double-stranded regions, G and C were the most frequently modified nucleotides. Furthermore, bulged bases, G-A- and G-U-wobble pairs were also highly susceptible to modification. The pattern of cross-linking was altered when 16S rRNA was photolabeled in its complex with ribosomal proteins. Unexpectedly, free 16S rRNA appeared to be less susceptible to cross-linking than the 16S rRNA in the 30S ribosomal subunit (>75% of the photolabel linked to rRNA). Furthermore, there were few additional alterations when ABA was cross-linked in complex C (Figure 3). This seems contrary to two previous reports which suggested that polyamine binding to rRNA is unaffected by ribosomal proteins (9,74). With regards to ribosomal function, spermine photolabeling increased the efficiency of AcPhe-tRNA binding to poly(U)-ribosome complexes, but decreased its ability to recognize and reject near cognate aminoacyl-tRNA. However, the catalytic activity of the reconstituted complex C was unaffected upon photolabeling. The authors succeeded to locate spermine binding sites in 16S rRNA regions implicated in several important ribosomal functions, including tRNA and subunit association, as well as cognate tRNA recognition (106).

When probing intact *E. coli* ribosomes greater than 57% of the photo-probe was cross-linked to rRNA (105). In the entire 23S rRNA (except for the terminal nucleotides 2870–2904), 54 cross-links were located, grouped into 37 independent regions. Again, both helices and single-stranded regions were found among the cross-links, with a preference for non-paired nucleotides (65%). Here, most of the labeled nucleotides in the single-stranded regions were A and U (75%). In contrast, cross-linking in double-stranded regions was distributed equally among the 4 nt, with a slight preference for pyrimidines (60%). Furthermore, competition experiments utilizing mono- and divalent ions, as well as the natural polyamines, suggested that ∼60% of the identified sites were in fact *bona fide* polyamine binding sites. Notably, most of the polyamine binding sites in the 23S rRNA were involved in prominent ribosomal functions. Additional functional studies utilizing 50S subunits, with and without prior ABA-spermine cross-linking, implicated an essential role for spermine in facilitating both peptide bond formation and tRNA-translocation efficiencies (105).

The polyamine binding sites close to the PTase center of 23S rRNA (domain V) were examined after photolabeling of the full ‘complex C’ with ABA-spermine (107,110). Here, the region encompassing the PTase loop was highly susceptible to cross-linking. In this central loop region (∼170 nt), where several antibiotics are known to bind, over half of the 15 cross-linking sites identified were again adenine residues, mostly in single-stranded regions (107,110). These sites did not always coincide with metal binding sites (107,110). Furthermore, as seen with dimethyl sulfate (DMS) probing, the central loop of the V domain underwent structural alterations upon cross-linking, forming ‘an apparent open tertiary structure’ (110). Polyamine cross-linking sites in the V domain coincided with (or were proximal to) nucleotides implicated in spiramycin, blasticidin S and chloramphenicol (CAM) binding (107,110). Indeed, polyamines have been suggested to alter antibiotic function by facilitating (CAM, blasticidin S) or restricting (spiramycin) their binding to the ribosome.

### tRNA

tRNAs are 70–95 nt in length and contain numerous conserved structural features including acceptor, anticodon, D- and T-stems as well as three/four loops, one of which is highly variable (the ‘variable’ loop). In addition to the natural bases, tRNAs also carry a number of modified nucleotides, for example the 4-thiouridine (s^4^U) residue(s) at the junction of the D and acceptor arms (11,101,111). Polyamines play an important role as an additive in tRNA crystallization (112) and for tRNA function (103). Studies carried out on tRNA–polyamine interactions prior to 1976 have been comprehensively reviewed by Sakai *et al.* (111). Their review also covers the impact of polyamine binding on the physical properties and the chemical reactivity of the s^4^U residue(s), which are located in a structural region thought to be stabilized by spermidine. Here, polyamines were shown to bind in the vicinity of the D-stem and potentially stabilized this region, resulting in specific effects on the tRNA conformation. The review also described the identification of discrete low-affinity and high-affinity binding sites for spermidine on tRNA^Phe^ as well as mixed tRNA from yeast. High affinity sites bound two to three molecules of spermidine per tRNA with association constants (*K*_a_’s) in the range of 6 × 10^4^ M^−1^. The remaining low affinity sites displayed *K*_a_'s of 5.4 and 7.6 × 10^3^ M^−1^, for tRNA^Phe^ and mixed tRNA, respectively. Of particular relevance, the review also summarizes evidence for the non-cooperatively of polyamine–tRNA binding; the equivalence of ethidium and spermidine in interacting with tRNAs and the functional equivalence of Mg^2+^ and polyamines for tRNAs (111). Below we discuss literature on polyamine–tRNA binding post 1976.

#### Structural studies using NMR spectroscopy and X-ray crystallography

The interaction of polyamines with tRNA has been studied at high resolution using NMR spectroscopy (19,78,103,113–115) and X-ray crystallography (116–119). Early studies utilizing ^1^H NMR spectroscopy revealed that spermine binding to tRNA (similarly to RNA homo-polymers: see the ‘Synthetic RNA models’ section) results in the loss of resonances of the ribose 2′-OH proton (78). Subsequently, Frydman *et al.* used ^13^C NMR spectroscopy to investigate the binding of isotopically labeled spermidine to mixed tRNA. Approximately 14 binding sites for spermidine were detected per tRNA equivalent. Spermidine at 11 of the binding sites was displaced by Mg^2+^ or K^+^ ions, leaving three spermidine molecules stably bound. Analysis of C-resonances adjacent to N-atoms suggested that primary amines were more mobile conformationally than secondary amines: hence the latter were more strongly bound. Interestingly, isotopically labeled spermidine could be displaced by unlabeled spermine, but not by putrescine, suggesting selectivity for polyamine binding (19). Data from studies of ^15^N-labeled polyamines supported these findings (113). In a separate account, spermine–tRNA binding sites (yeast tRNA^Phe^) were identified by ^1^H–^1^H Nuclear Overhauser Effects between spermine and protons of the tRNA. The data suggested that spermine bound at the junction of the TΨC (T-loop) and D-loops, a critical region for correct tRNA function (120).

**Figure 4.**
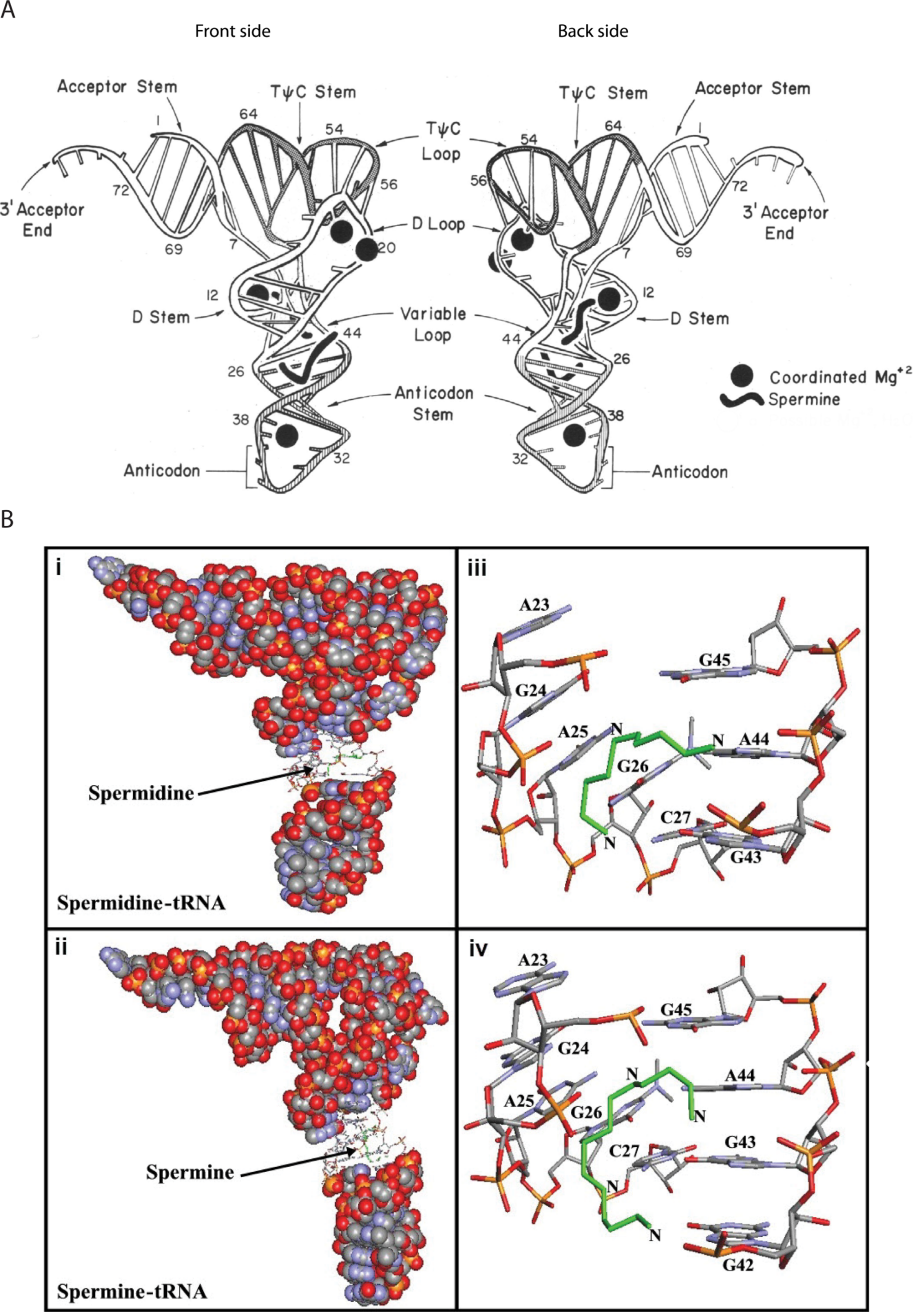
(**A**) Schematic diagram showing two sides of yeast tRNA^Phe^. The positions of two spermine molecules and four magnesium ions are shown as solid black figures. Front side: spermine molecule extends from the D-stem into the anticodon stem. Back side: spermine molecule binds at the junction of the D and acceptor stem, in close proximity to the variable loop. Figure edited from (116). (**B**) Best conformations for polyamines docked to tRNA (PDB entry 6TNA). Green—the polyamines. tRNA in sphere-filling model with (i) spermidine and (ii) spermine binding sites in sticks. Binding sites for (iii) spermidine and (iv) spermine represented in sticks with the corresponding base residues. Figure edited from (129).

The structures of tRNA^Phe^-spermine complexes have been resolved by X-ray crystallography (116–118). Two binding sites for spermine were identified by Quigley *et al.* (116) (Figure 4A). One spermine is shaped like a fish hook in the deep groove of the double helix and extends from one end of the D-stem into the anticodon stem. It affects the orientation of the anticodon stem, bringing the two helical stands together by ∼3 Å at the major groove, and alters the helical axis of the anticodon stem. The second spermine binds near the variable loop at the junction of the D and acceptor stem (116). In an independent study a third spermine was unambiguously located in the major groove of the helix formed by the acceptor stem and the T-stem (117). The approximate positions of these three spermines were predicted to be amongst the most optimal in tRNA^Phe^-spermine complexes (121). In a recent study, a single spermine was also located in the major groove of the T-stem and loop of tRNA^Phe^, with probable contacts to nucleobases, phosphates and water molecules and sandwiched between two tRNA molecules of a dimer (118).

#### Site directed cleavage studies

Cross-linking, as described earlier for rRNA, and site-directed cleavage reagents has been used by several groups to map the positions of polyamine binding sites on tRNA. A spermine-linked photosensitive RNA-cleaving probe possessing an aryldiazonium group (SperN_2_^5+^: Figure 1) was used to uncover three binding sites on yeast tRNA^Asp^ which were confirmed through competition with wild-type spermine (122). Polyamines were bound predominantly at regions of the tRNA structure where the negative charge density is predicted to be highest (121). The strongest interaction with spermine was where the variable loop faces the junction between the acceptor- and the D-stems, consistent with an earlier report (116). The additional polyamine binding sites were located in the T-loop in the heart of the tRNA structure, and in the deep narrow groove joining the anticodon and D-stem, close to a spermine binding site seen in the tRNA^Phe^ crystal structure (116). For the latter, multiple cleavage events were seen, possibly indicating mobility of the polyamine between isoenergetic overlapping binding sites or a rotational flexibility of the molecule (122). Of note, the polyamines exhibited comparable affinities for these binding sites, suggesting that at least in these experiments the polyamine binding sites in tRNA show little selectivity for the polyamine structure. Furthermore, at high probe concentration the majority of the phosphate backbone of tRNA^Asp^ was cleaved. However, three regions remained un-cleaved, including one where Mg^2+^ is known to coordinate in tRNA^Phe^ (116), suggesting that these regions are not prone to polyamine binding.

Other chemical tools have been used in cleavage experiments to reveal sites of interaction between polyamines and tRNAs. In one study spermine-imidazole conjugates (Figure 1, Sp-Im) were employed. A pattern in agreement with that published earlier was obtained (122). Here, all cleavages occurred after pyrimidine residues and for the most part, within pyrimidine-A sequences, especially CpA. In this study, however, the investigators did not confirm the binding sites using unlabeled spermine (123).

Two cross-linker analogs of spermine—ABA-spermine and ANB-spermine (containing three rather than four positive charges) (Figure 1)—were used to map polyamine binding sites in AcPhe-tRNA, both in its free form and bound at the P-site of the *E. coli* poly(U)-ribosome complex (109). The sites of cross-linking were determined through nuclease probing and were confirmed by competition experiments with up to 250-fold unlabeled spermine, which inhibited up to 80% of the cross-links. The authors described a strong cooperative binding of the probes to free tRNA^Phe^. Favored binding sites on AcPhe-tRNA in free solution were the deep pocket formed by the nucleotides of the D-stem and the variable loop, as well as the anticodon stem and the T-loop. These matched closely to spermine binding sites found in yeast tRNA^phe^ by X-ray crystallography and earlier cross-linking studies (116–118,122). In addition, gel shift assays suggested that cross-linked tRNA^Phe^ possessed a larger inter-stem angle between the anticodon and acceptor stem than the corresponding unlabeled tRNA^Phe^, consistent with chemical probing experiments which showed conformational changes in the structure. In general, an increase in the degree of photolabeling was observed when AcPhe-tRNA was bound to poly(U)-ribosome complexes. This suggests that the probes were bound more strongly to complexed AcPhe-tRNA than AcPhe-tRNA alone. Finally, the functionality of the photolabeled AcPhe-tRNA-poly(U)-ribosome complex in peptide bond formation was evaluated. Here photolabeling was shown to affect peptide bond formation activity depending on whether the AcPhe-tRNA-poly(U)-ribosome complex was first formed and then irradiated in the presence or absence of translation factors: in the absence of translation factors activity was reduced, whereas it was stimulated when photolabeled in the presence of translation factors (109).

#### Miscellaneous techniques

Other techniques have been used to investigate polyamine–tRNA interactions, including Fourier transform infrared spectroscopy (FTIR), analysis of UV differential melting curves, electron spin resonance (ESR) spectroscopy, RNase foot-printing, equilibrium dialysis, molecular modeling, cleavage assays, competition assays and CD (81,96,124–135). Selected examples are discussed below.

Formation of the rat liver Ile-tRNA complex by lle-tRNA synthetase is selectively stimulated by spermidine, whereas the formation of other Ile-tRNA complexes such as those for *E. coli* and torula yeast were not (96,125,134). Binding studies and RNase foot-printing suggested that physical interactions between spermidine and rat liver tRNA^Ile^ are important for this effect. Here spermidine-mediated conformational changes in the acceptor and anticodon stems were shown to be specific for rat liver RNA^lle^, and therefore of particular interest (125,134). Data from CD experiments later highlighted a G–G mismatch in the stem of the tRNA^Ile^ as required for a stabilization of the accepter stem by spermidine (96); the same stem of torula yeast RNA^lle^, which does not contain a mismatch, was not stabilized by the polyamine (96). The sites and modes of spermidine binding to rat liver lle-tRNA^Ile^ have been investigated using molecular modeling, density functional theory calculations and molecular dynamics simulations (128). The authors proposed that under physiological conditions both a terminal NH_3_^+^ and the adjacent secondary NH_2_^+^ of spermidine form H-bonds with the RNA bases. In contrast, the remaining NH_2_^+^ interacts ionically with the phosphate backbone. H-bonds between spermidine and the mismatched G residues in the acceptor stem of tRNA^Ile^ were suggested to be responsible for spermidine-induced RNA structural alterations (128).

UV differential melting curves used to investigate the effect of cationic binders on the thermal stability of tRNA revealed two clearly separated melting transitions specifically for yeast tRNA^iMet^ (127). The authors assigned the first and second transitions to the unfolding of the tertiary and secondary structures of the tRNA, respectively. Here, spermine and spermidine were inferred to be less capable than Mg^2+^ of stabilizing the former melting transition. On the other hand, the polyamines were slightly better at stabilizing the second melting transition. The authors proposed that ‘elongated cations’ such as polyamines are better suited to bind to clefts in the secondary structure rather than to pockets in the tertiary structure, unlike spherical Mg^2+^ which binds both (127). In a quest for more insight into spermine–tRNA interactions, the naturally occurring modified base, N^6^-isopentenyladenosine (i^6^A), positioned adjacent to the anticodon (i^6^A37) in yeast tRNA^Tyr^ was spin-labeled. ESR was then used to monitor spermine-mediated effects on the label as a function of temperature. The polyamine induced a stabilizing effect on a ‘pre-melting’ transition. This suggested that the polyamine stabilizes a structure(s) in the vicinity of i^6^A37 (103,111). Further investigations into the stabilization of this pre-melting transition revealed sequential binding of multiple spermines to the tRNA. Binding of the fifth spermine molecule to a site rather distant from the i^6^A37 residue was shown to be responsible for the aforementioned stabilization. Using Mn^2+^ as a paramagnetic substitute for Mg^2+^, the binding of spermine was observed to create new strong binding sites for divalent cations (103,126,135).

Using FTIR, capillary electrophoresis, and molecular docking, Ouameur *et al.* attempted to identify points of contact between polyamines and yeast tRNA (129). At low concentrations of spermine and spermidine a hypochromic effect in the IR spectra indicated that both polyamines were capable of stabilizing the tRNA structure. Sites of preferential binding were the nucleobases (G-N^7^/O^6^, U-O^2^/O^4^, A-N^3^) and the ribose moieties (Figure 4B). At high concentrations of putrescine as an example, the hyperchromic effect implied that helix destabilization was occurring: interactions with the riboses, G-N^7^/O^6^ and U-O^2^ became more pronounced, and additional contacts including A-N^7^, the phosphate-ribose backbone and various hydrophobic interactions were noted (129). This coincided with other spectroscopic and biochemical analyses, suggesting that strong polyamine binding takes place when the polyamine–tRNA ratio is lower than 4:1, whereas weaker and probably less specific interactions occur at higher polyamine–tRNA ratios (113). Notably, no major tRNA conformational changes were observed, suggesting the tRNA remains in an A-form structure in these polyamine–tRNA complexes. Using capillary electrophoresis the affinities of polyamines for tRNA were calculated to be 8.7 × 10^−5^ M, 6.1 × 10^−5^ M and 1.0 × 10^−5^ M for spermine, spermidine and putrescine, respectively (129). These affinities are in the range of the equilibrium dissociation constants calculated for polyamine binding to tRNA^Phe^ and tRNA^Glu^ based on equilibrium dialysis binding data gathered under low ionic strength conditions (130). Furthermore, positive cooperativities of binding were detected (Hill coefficients: 1.42, 1.14, 1.12, respectively). Docking studies indicated that spermidine, spermine and putrescine all bind to a common purine-rich site in the major groove, extending from the D-stem into the anticodon stem (Figure 4B), in addition to the T-loop (129).

Taking the lessons from these studies together, it is clear that specific polyamine dependencies are likely decisive for the proper folding of particular tRNA species at various stages of protein synthesis, and that chelated polyamines and divalent ions play distinct structural roles. The observations put to rest the simplistic notion that polyamines are simply organic cations, analogous to Mg^2+^ and K^+^.

### Ribozymes

Ribozymes are RNA molecules that predominantly catalyze their own cleavage or that of other RNAs (136,137). They can generally be categorized into self-splicing group I/II introns, RNase P, self-cleaving regions of viral RNA, plant satellite RNAs and rRNA (101). The effect of metal ions on ribozyme activity has been thoroughly documented (138–140). The first report of polyamines having a role in ribozyme catalysis appeared in 1986, when spermidine was reported to promote the production of monomeric RNA from dimeric STobEV RNA (141). Since then, polyamines have been shown to function not only as ribozyme activators but also as inhibitors of ribozymes. For example, several studies have demonstrated that spermidine and/or spermine can promote ribozyme catalysis in the presence of Mg^2+^ or other divalent ions, with examples including the hairpin ribozyme (142–145), selected hairpin ribozyme variants (146), the hepatitis delta virus ribozyme (147), RNase P (148), group I introns (149–151), a class II mitochondrial intron (152), the Hammerhead ribozyme (153,154) and also on some occasions the Neurospora VS RNA (146,155–157). On the other hand, spermidine has been reported to inhibit ribozyme activity, particularly at high concentrations (144,146,158,159). Polyamines have also been shown to function as ribozyme-folding or cleavage modulators in the absence of Mg^2+^ and in some cases in a manner differing to that of divalent cations (144,148,153,154,156,160–165). On a structural note, in recently published crystal structures of the *Oceanobacillus iheyensis* group IIC intron (self-splicing metalloribozyme) two putative polyamine binding sites were identified (140). One of these sites is proximal to a phylogenetically conserved kissing loop, while the other is proximal to a T-loop. Binding of polyamines to the latter site displayed an RNA interaction pattern resembling the binding of basic amino acids to T-loops in certain ribonucleoproteins, suggesting polyamines may bind with RNA at sites predicted to interact with proteins (140). Summarizing, the sequence and structure of the ribozyme, as well as its mechanism, likely determine the roles of polyamines in ribozyme function.

## SUMMARY AND OUTLOOK

In spite of their critical roles in many cellular processes, the mechanistic workings of polyamines are rarely investigated. Indeed, one can argue that polyamines still reside in ‘the box-room of biochemistry’ (166). One reason is the inherent difficulties in designing and interpreting experiments involving these small ubiquitous molecules, as evidenced by many incongruences in the literature associated with the nature of polyamine–RNA interactions. The main reason, however, is likely their lack of molecular complexity which, paradoxically, implies functional simplicity: polyamines are still mostly seen as poly-cations, despite the well-known non-interchangeability of even the simplest cationic species, Mg^2+^ and Ca^2+^ in biological systems (166).

As for metal ions (167), two distinct modes of binding to RNA can be expected for polyamines (105,111): an orthodox non-specific manner, where polyamines diffuse within a restricted volume around the nucleic acid or its hydrated environment and a site-specific mode, where it is chelated in a defined binding pocket via direct interactions with distinct nucleic acid residues. In the latter case, these interactions will be governed by the polyamine structure, its protonation state, the RNA structure and sequence and the ionic environment. Furthermore, polyamines can be covalently modified, for example by oxidation, acetylation or conjugation (references cited in (55,67,168)), potentially increasing the ways in which they might interact with RNA. Our survey of the literature has highlighted many examples of polyamines interacting with RNA in a sequence/structure-selective manner (often with functional consequences). For example, upon probing polyamine binding sites with cross-linking reagents, only a small fraction of the residues in the rRNA targets were cross-linked and large regions of the rRNA remained unmodified. Importantly, regardless of the chemical probe used, the cross-links in both rRNAs and tRNAs were predominantly at A and/or U residues in weakly structured regions, at bulges and wobble base pairs. Where investigated, a majority of the polyamine binding sites were distinct from those that bind Mg^2+^. In addition, preferential binding sites for polyamines are important for specific functions of rRNA, tRNA and antibiotics; furthermore cross-linking also affected these functions. Several *in vitro* and *in vivo* investigations revealed the bulged nucleotides of stems in mRNA and snRNA, and a mismatch in a tRNA stem, as required features for stem stabilization by spermidine. Spermidine-specific effects included stimulating initiation of mRNA translation, modifying the viral protein binding capacity of a snRNA and charging the tRNA. Where investigated, Mg^2+^ could not substitute for the polyamine, providing strong evidence that polyamines do indeed interact with RNA in specific fashions and that the interactions direct and/or modulate RNA functions.

In three independent studies of tRNA the same polyamine binding sites were identified using three unrelated techniques. These sites are the anticodon stem, the region between the D and acceptor stem and the T-loop. It is highly likely these three polyamine-bound binding sites play important roles in the general function of tRNAs. Furthermore, a unique binding site for spermidine was found in the acceptor stem of rat liver tRNA^Ile^ at a sequence motif absent in other tRNAs. As the polyamine stimulates rat liver Ile-tRNA complex formation but not other Ile-tRNA complexes (i.e. *E. coli* tRNA^Ile^), this is evidence for functional polyamine–tRNA binding events that are specific for a particular tRNA.

Taken together, the evidence that polyamines directly regulate key RNA functions is wide-ranging and substantial. Thus, natural adjustments in the cellular concentrations of specific polyamines have the potential to control this regulation by shifting the dynamics of RNA structure. To date, the primary focus of investigations has been the protein synthesis pathway. However, polyamines have also been shown to interact with viral RNAs (169,170). Additionally, polyamines modulate a variety of additional processes, such as splicing and helicase activity, all of which could involve direct interactions with RNAs. Hence, mechanistic investigations into polyamine–RNA regulation on the transcriptome scale *in vivo* (171–174) or even *in vitro* (as recently performed for Mg^2+^ (173)) would be highly informative. We are looking forward to a new era of exploration in the polyamine–RNA field.
